# An incidental finding of chronic lymphocytic leukaemia in a patient with pulmonary tuberculosis

**DOI:** 10.4102/sajid.v35i1.218

**Published:** 2020-12-10

**Authors:** Victoria R. Parker, Jaclyn A. Bennet, Ian M. Sanne

**Affiliations:** 1 The Clinical HIV Research Unit, Department of Internal Medicine, School of Clinical Medicine, Faculty of Health Sciences, Wits Health Consortium, University of the Witwatersrand, Johannesburg, South Africa

**Keywords:** tuberculosis, leukaemia, chronic lymphocytic leukaemia, infectious diseases, haematology

## Abstract

In this case report, we described a 48-year-old human immunodeficiency virus-negative man with pulmonary tuberculosis (TB) who had an incidental finding of concurrent chronic lymphocytic leukaemia. We highlighted the importance of considering other differential diagnoses when patients present with TB or haematological malignancies. We also discussed the difficulties in diagnosis when both of these conditions co-exist and how this affected treatment and management.

## Introduction

Tuberculosis (TB) is a major health problem worldwide. It is ‘one of the top 10 causes of death and the leading cause from a single infectious agent (above human immunodeficiency virus [HIV] and/or acquired immunodeficiency syndrome [AIDS])’.^1^ According to the World Health Organization (WHO), approximately 10 million people worldwide suffered from TB in 2018.^1^ Eight countries accounted for two-thirds of the global total, of which South Africa accounted for 3%.^1^ It is estimated that about one quarter of the global population is infected with latent TB which could lead to active disease.^1^

Immunocompromised patients, including those with haematological malignancies, are at an increased risk of developing active TB and these patients have an increased mortality risk.^2,3^ The estimated relative risk of developing active TB in patients with haematological malignancies is between 2 and 40 times that of the general population.^2^ This vast range can be attributed to the use of different diagnostic criteria, different patient characteristics and the prevalence of disease in different areas and countries.^4^ The symptoms that present during active TB and various haematological malignancies are similar, which may lead to misdiagnosis or a delay in diagnosis of the primary cause of disease. In this article, we present the case report of a patient who was diagnosed with pulmonary TB with an incidental finding of concurrent chronic lymphocytic leukaemia (CLL).

## Case presentation

On 26 November 2019, a 48-year-old, HIV-negative man presented to the Primary Health Care Facility in Johannesburg, South Africa, with symptoms suspicious of TB. His sputum was collected and sent for GeneXpert testing. This confirmed a diagnosis of rifampicin-sensitive TB. On 28 November 2019 he was referred to the Clinical HIV Research Unit for screening for an observational study called TB Sequel.^5^ The TB Sequel project’s aim is:

[*T*]o advance the understanding of the clinical, microbiologic, environmental and host immune factors affecting the long-term sequelae of pulmonary TB; to determine occurrence of reversible and irreversible costs and socioeconomic consequences for TB patients and health care systems; and to facilitate novel interventions to restore and preserve overall health, well-being and financial protection in patients with TB.^5^

At the screening visit, the patient complained of a 3 month history of cough, fatigue, night sweats, body weakness, loss of appetite, loss of weight, chest pain, shortness of breath, nausea and vomiting. He also complained of an intermittent fever since February 2019. He had no significant family history of malignancy.

On examination, he was generally wasted but apyrexial and the rest of his vital signs were normal. He had cervical and axillary lymphadenopathy and a hepatomegaly (without splenomegaly). His chest examination revealed scattered lung crackles. He was managed by his Primary Health Care Facility according to the National Tuberculosis Management Guidelines and was thus started on treatment for rifampicin-sensitive TB, rifafour (rifampicin, isoniazid, pyrazinamide and ethambutol), pyridoxine and vitamin BCO on the same day.^6^

He was enrolled onto the TB Sequel study on 02 December 2019 and routine baseline investigations were performed.^5^ His chest X-ray showed bilateral infiltrates, left hilar lymphadenopathy and a right pleural effusion, with approximately 80% of his lungs affected. His baseline bloods, along with repeated blood results (see Table 1), showed abnormalities in the full blood count, specifically of the white blood cells. His blood results were suggestive of a lymphoproliferative disorder which required further workup.

**TABLE 1:** Blood results from the patient’s clinic and hospital notes.

Test: Full blood and differential count	Date and values		Reference range
	Monday, 02 December 2019	Wednesday, 11 December 2019	
White cell count	150.43 x 10^9^/L	123.90 x 10^9^/L	3.92–10.4 x 10^9^/L
Haemoglobin	13.0 g/dL	12.2 g/dL	13.4–17.5 g/dL
MCV	80.6 fL	83.3 fL	83.1–101.6 fL
MCHC	30.7 g/dL	30.4 g/dL	33.0–35.0 g/dL
Platelet count	584 x10^9^/L	503 x 10^9^/L	171–388 x 10^9^/L
Neutrophils	7.52 x 10^9^/L	12.39 x 10^9^/L	1.60–6.98 x 10^9^/L
Lymphocytes	141.37 x 10^9^/L	95.96 x 10^9^/L	1.40–4.20 x 10^9^/L
Monocytes	1.50 x 10^9^/L	1.21 x 10^9^/L	0.3–0.8 x 10^9^/L
Eosinophils	0.00 x 10^9^/L	1.21 x 10^9^/L	0.00–0.95 x 10^9^/L

MCV, mean corpuscular volume; MCHC, mean corpuscular haemoglobin concentration.

The patient was admitted to a tertiary public hospital in Johannesburg on 11 December 2019 for further investigation. His full blood count, differential count and peripheral smear upon admission showed a profound absolute lymphocytosis, comprising predominantly small mature forms, with occasional smudge cells and large granular lymphocytes. Approximately 3% of these were larger more primitive cells with features in keeping with prolymphocytes. No increase in blasts was noted. No other secondary causes for eosinophilia and thrombocytosis were discovered and both were regarded as reactive in nature. His lactate dehydrogenase (LDH) from 12 December 2019 was 262 U/L (reference range: 100–190 U/L). His renal function and liver function tests were normal.

A bone marrow aspirate and trephine biopsy was performed on 12 December 2019 and referred to the National Health Laboratory Service for routine analysis. The report described a hypercellular bone marrow with extensive diffuse infiltration by small mature lymphocytes. Fluorescence *in situ* hybridisation (FISH) performed on the bone marrow aspirate sample confirmed that all screened cells were negative for (1) deletion of the *tumour protein p53 (TP53)* and *ATM serine/threonine kinase* (*ATM)* genes, (2) deletion of the long arm of chromosome 13 (*13q14.3)* and (3) trisomy of chromosome 12. Flow cytometry immunophenotyping performed on the bone marrow aspirate showed a population (‘81%) of clonal (kappa light chain restricted) B-cells, which expressed the following markers: CD19+, CD5+/++, CD20++, CD23 dim/+, CD45+/++, CD22+, CD38 and possible dim FMC7. CD10 was not expressed.

These morphological and immunophenotypical findings were in line with a diagnosis of CLL.

A staging computed tomography (CT) scan was performed on 18 December 2019, which showed an extensive bilateral tree-in-bud pattern, and a right-sided pleural effusion, both are suggestive of active pulmonary TB. Signs of pulmonary artery hypertension as well as supraclavicular, mediastinal and intra-abdominal lymphadenopathy were also noted. Furthermore, a thrombus in the inferior vena cava (IVC) was seen, starting from the level of the renal veins and extending distally. Although a hepatomegaly was found on clinical examination, the CT scan confirmed a normal liver and spleen, with no focal lesions. An echocardiogram was performed on 19 December 2019, which was normal.

Based on all of the above results, the patient was classified as stage I under the Rai classification and stage B using the Binet classification of CLL.^7,8^ He had both good (Rai I) and bad (male sex, Binet B, diffuse bone marrow appearance, CD38 expression and raised LDH) prognostic indicators, which would affect his management.^9^

The patient was initially anticoagulated and monitored by the tertiary public hospital’s coagulation clinic for the IVC thrombus and subsequently referred to the oncology unit at Charlotte Maxeke Johannesburg Academic Hospital for further management of his CLL. As cure is rare in CLL, treatment is mainly symptomatic and the risk–benefit analysis of available treatments should be considered on a case-by-case basis.^9^

### Ethical consideration

Ethical approval to conduct the study was obtained from the Human Research Ethics Committee (Medical) of the University of the Witwatersrand (Reference number: R14/49, Protocol No: M1911204) on 29 January 2020. Informed consent for publication of this case report was obtained from the patient.

## Discussion

This case report of an HIV-negative man, who was initially diagnosed with TB and then was subsequently diagnosed with a concurrent haematological malignancy, highlights some important factors in the diagnosis and management of both of these conditions.

We will start by discussing the differential diagnoses of an elevated white cell count in the setting of an active infection, like TB. We will then discuss how various haematological malignancies result in immunocompromise, which increases the risk of TB. We will also discuss the difficulties in the diagnosis of both of these diseases because of symptom overlap and atypical presentations. Lastly, we suggest that where economically viable, appropriate workups should be performed to investigate for other diseases.

When faced with a patient who has TB, or another active infection, and a markedly elevated white cell count, it is important to consider a range of causes for leukocytosis. A rare cause of a markedly elevated white cell count is a leukemoid reaction.^10^ This is a leukocyte count above 50 × 10^9^/L that is not caused by leukaemia.^10^ Whilst this condition is rare, it is essential to differentiate it from a myeloproliferative disorder, as a leukemoid reaction is a diagnosis of exclusion.^10^ A retrospective cohort study performed at a tertiary hospital in Brazil found that 60% of the 267 cases who had white blood cell counts of more than 50 × 10^9^/L were caused by a haemapoeitic neoplasm.^10^ The other 40% of cases were true leukemoid reactions, with infections being the leading cause, followed by non-haematopoeitic neoplasms and other causes.^10^

An accurate definitive diagnosis of the cause of the elevated white cell count should be made timely. A case series documented three cases of delayed diagnosis of myeloproliferative disorders, which were mistaken as leukemoid reactions in the setting of mycobacterial infection.^11^ Inversely, it is important not to jump too quickly to a diagnosis of a malignancy without definitive proof, especially in the setting of infection. This was illustrated in a case study of a 22-month-old boy who was initially misdiagnosed as having leukaemia, but his leukocytosis was later found to be because of a leukemoid reaction, secondary to a necrotizing pneumonia. This caused unnecessary emotional harm to the patient and his family.^12^

Typically, a leukemoid reaction’s leukocytosis predominantly involves neutrophils of a polyclonal nature.^12^ In our patient, however, the profound absolute lymphocytosis and evidence of clonal B cell expansion led to the diagnosis of CLL, therefore excluding a leukemoid reaction as the cause of leukocytosis.

Immunocompromised patients are at an increased risk of developing infections such as TB. They are also at an increased risk of latent TB becoming active TB.^13^ The incidence of TB in patients with haematological malignancies can be attributed to T-cell immunodeficiency as a result of the malignancy itself or its treatment.^4^ Patients with haematological malignancies have an increased risk of developing TB; however, this risk varies depending on the type of haematological malignancy and various studies have shown conflicting data.^2^ A retrospective study performed in Taiwan between 1996 and 2009 found that the overall incidence of TB in patients with haematological malignancies was 1.78% and this was the highest in patients with acute myeloid leukaemia.^14^ Another study performed in Rio De Janeiro between 1990 and 2000 found that the prevalence of TB in patients with haematological malignancies was 2.6% and this was the highest in patients with CLL.^4^

Because of the high prevalence of TB in South Africa, it is often near the top of a differential diagnosis list when treating patients presenting with constitutional symptoms, like those seen in our patient. A retrospective study performed in KwaZulu-Natal found that 18 out of the 21 patients (85.7%), who were found to have lymphoma, had been treated for TB in the preceding year before the diagnosis of lymphoma was made.^15^ Most of these patients reported that they were treated for TB based on history and examination alone.^15^ They reported little or no improvement in symptoms on treatment.^15^ This illustrates the importance of an accurate diagnosis using certified laboratory tests along with history and examination findings.

It can be very difficult to differentiate between active TB and various haematological malignancies because the symptoms present in both diseases are often very similar. Active TB often presents with some or all of the following symptoms: cough, fever, weight loss, night sweats, haemoptysis, chest pain and fatigue. Some of these symptoms are also common in haematological malignancies, which make an accurate diagnosis difficult. This was demonstrated in a retrospective study performed at a cancer centre in the United States of America, which found that one-third of patients who were referred to the unit for a suspected malignancy between 2006 and 2014 were diagnosed with active TB with no co-morbid cancer.^16^

Another common finding in both diseases and present in our patient is the presence of lymphadenopathy. In the absence of a definitive diagnosis, the cause of lymphadenopathy should be investigated. An article by Antel and Verburgh describes the difficulties in sampling and diagnosing lymphadenopathy in various areas of the body and highlights the mistakes often made by clinicians, leading to misdiagnosis or late diagnosis of malignancies.^17^ The article provides a suggested diagnostic and management approach for patients with lymphadenopathy in various anatomical areas of the body.^17^ It suggests that empiric TB therapy should only be given to those patients who have lymph nodes not amenable to biopsy.^17^ In these specific cases, close monitoring and follow-up are necessary to ensure treatment is effective and no other underlying co-morbidity or disease has been missed.^17^

Another issue of concern with both these conditions is that immunocompromised patients, including those with haematological malignancies, with active TB often have atypical radiological findings that could confuse the treating clinician.^3^ These may include fewer cavities or calcification, bilateral consolidation mainly affecting the lower lung fields and mediastinal or hilar lymphadenopathy.^3^ It is therefore vitally important to consider the diagnosis of active TB in all these patients and investigate appropriately. As delayed diagnosis and treatment of TB could lead to further transmission of TB to other patients, healthcare workers and the general population, prompt diagnosis and prompt treatment of active TB are imperative.

As mentioned previously, it may be difficult to ascertain which symptoms are caused by a haematological malignancy and which are caused by TB as both of these overlap. South Africa has a high burden of TB and for this reason, patients who present to their local clinic with symptoms suggestive of TB are tested. If TB is found, these patients are treated appropriately. As part of the National Tuberculosis Management Guidelines, baseline blood tests are not routinely performed in these patients.^6^ Therefore, had this patient not been screened for a research study, it is likely that the diagnosis of CLL would have been delayed. To avoid future cases like this, it could be argued that baseline bloods should be drawn from ‘certain patients’ with TB. The challenge would be to determine ‘which patients’ and to determine the balance between the economic impact of baseline blood tests versus the benefit of screening for other diseases and disorders (such as haematological malignancies).

## Conclusion

As described in this study, there is a significant overlap between symptoms present during active TB and constitutional symptoms of haematological malignancies. It is therefore important to investigate thoroughly, follow diagnostic algorithms and use sound clinical judgement when treating these patients. In countries like South Africa, where the incidence of TB is high in the general population, it is important to treat patients with TB appropriately and timely. However, not all symptoms indicate TB. Patients need to be worked up appropriately and, where economically viable, the possible presence of other conditions should be investigated.
